# Abnormal Expression of BTLA and CTLA-4 Immune Checkpoint Molecules in Chronic Lymphocytic Leukemia Patients

**DOI:** 10.1155/2020/6545921

**Published:** 2020-07-28

**Authors:** L. Karabon, A. Partyka, L. Ciszak, E. Pawlak-Adamska, A. Tomkiewicz, A. Bojarska-Junak, J. Roliński, D. Wołowiec, T. Wrobel, I. Frydecka, A. Kosmaczewska

**Affiliations:** ^1^Department of Experimental Therapy, Hirszfeld Institute of Immunology and Experimental Therapy, Polish Academy of Sciences, Wroclaw, Poland; ^2^Department and Clinic of Urology and Oncologic Urology, Wroclaw Medical University, Wroclaw, Poland; ^3^Department of Clinical Immunology and Immunotherapy, Medical University of Lublin, Lublin, Poland; ^4^Department and Clinic of Haematology, Blood Neoplasms, And Bone Marrow Transplantation, Wroclaw Medical University, Wroclaw, Poland

## Abstract

Chronic lymphocytic leukemia (CLL) is characterized by the peripheral accumulation of neoplastic B cells and is frequently complicated by the systemic immunosuppression associated with an impairment in B and T lymphocyte activation. We hypothesized that the expression of immune checkpoint suppressors B and T lymphocyte attenuator (BTLA) and cytotoxic T lymphocyte antigen (CTLA-4) is disturbed in both lymphocyte subpopulations in CLL. The expression of CTLA-4 and BTLA mRNA was determined by real-time PCR, while CTLA-4 protein expression (surface or intracellular) was estimated in BTLA+ lymphocytes by flow cytometry. In CLL patients, we observed a higher gene transcript level of BTLA and CTLA-4 than in healthy individuals in both freshly isolated and PMA stimulated B and T cells. Remarkably, lower amounts of both inhibitory proteins were found in peripheral blood (PB) CLL B cells, whereas normal BTLA and elevated CTLA-4 were found in T cells. Consistently, there was a prevalence of CTLA-4+ cells within circulating BTLA+ T cells cells of patients confronting PB healthy cells. After *in vitro* stimulation, the only change found in CLL patients was a decrease in BTLA expression in B and T lymphocytes. In contrast, healthy lymphocytes responded more vigorously as regards the BTLA and CTLA expression with substantially higher frequency of CD69+ cells under the stimulating condition compared to corresponding cells from the CLL group. Our results indicate that CLL development is associated with the affected expression of BTLA and CTLA-4 checkpoint receptors in PB and its impaired expression might be associated with lowering of the threshold for B cell activation and proliferation, while upregulated CTLA-4 expression in CLL peripheral BTLA+ T cells may contribute to suppressed T cell effector functions. This hypothesis needs to be validated in future studies, which would allow us to explain how the increased or decreased expression of these molecules affects the cell function.

## 1. Background

Chronic lymphocytic leukemia (CLL) is the most common adult leukemia in western countries and is characterized by the gradual accumulation of mature B lineage-specific markers such as CD19, CD20, and CD23 and additionally the CD5 antigen in lymphoid tissues, bone marrow, and peripheral blood (PB). The clonal B cells generated in CLL might be acquired at the hematopoietic stem cell stage. The leukemic transformation is initiated by specific genomic alterations among others causing the deletion of specific microRNA genes and increasing the resistance of B cells against apoptosis (reviewed in [1]).

The discovery that malignant cells can evade the host immune systems by inhibiting T cells focused the attention on new therapeutic targets in cancer therapy—immune checkpoint inhibitors. In fact, the increased expression of the cytotoxic T lymphocyte antigen 4 (CTLA-4) molecule was found in the T cell compartment in CLL patients [2–4]. CTLA-4 blockade was associated with potent T cell proliferation in response to autologous and allogeneic CLL B cells, suggesting that this approach could represent a therapeutic opportunity to enhance an immune response against leukemia cells. However, as was shown by us and others, CTLA-4 protein expression in peripheral CLL cells is higher than that in normal B lymphocytes and positively correlates with better outcomes for CLL patients [5–8]. Our recent report clearly indicated that the response to the CTLA-4 blockade varied between CLL patients. For “high CTLA-4 expressed patients,” this approach induces prosurvival signals and is an unfavourable strategy for these patients, while patients with low CTLA-4 expression might benefit from CTLA-4 blocking therapy [8].

Another coinhibitory molecule, the B and T lymphocyte attenuator (BTLA), a member of the immunoglobulin superfamily providing inhibitory signalling via the T cell receptor (TCR) or the B cell receptor (BCR), is considered as a potential “immune checkpoint molecule” [9, 10]. BTLA is a type 1 membrane glycoprotein which in contrast to programmed cell death 1 (PD-1) and CTLA-4 binds to the herpesvirus entry mediator (HVEM), which is a member of the TNF receptor superfamily [11–13]. As demonstrated in *in vitro* studies, BTLA has a direct negative activity on T cell proliferation and cytokine production. BTLA-deficient T and B cells show enhanced proliferation in response to anti-CD3 and anti-IgM stimulation, respectively [11], while BTLA-deficient mice exhibit enhanced predisposition to autoimmunity [11]. In an animal model, BTLA expression was observed not only on CD4+ T cells and B cells but also on a wide range of hematopoietic cells, including CD8+ T cells, natural killer (NK) T cells, NK cells, macrophages, and dendritic cells (DC) [14, 15].

Literature data on human BTLA expression are limited. It was reported that BTLA is highly expressed on CD14+ monocytes and CD19+ B cells, constitutively on CD4+ and CD8+ T lymphocytes, and weakly on CD56+ NK cells [10]. It was shown that cross-linking BTLA with a monoclonal antibody (mAb) inhibits T cell proliferation, suppresses T cell activation, and produces the following cytokines: interferon- (INF-) *γ*, interleukin- (IL-) 2, IL-4, and IL-10, suggesting that BTLA-negative signals influence both Th1 and Th2 polarization [10].

BTLA plays an important role in the maintenance of T cell tolerance, as disturbances of the BTLA-HVEM pathway have been shown to be involved in the pathogenesis of neoplastic disorders [16], infections [17], and autoimmune diseases [14]. Aberrant expression of BTLA on B leukemic cells was also observed in CLL [16].

We hypothesized that expression of immune checkpoint molecules is disturbed either on B cell or on T cell populations. For verification of this hypothesis, we determined the expression levels of BTLA in CD3+ and CD19+ subpopulations of cells in CLL patients compared to those of healthy controls as well as to the coexpression of another inhibitory molecule CTLA-4 before and after *ex vivo* stimulation.

## 2. Materials and Methods

### 2.1. Patients

Patients were diagnosed based on criteria from the International Workshop on Chronic Lymphocytic Leukemia [18]. Patients' characteristics are presented in Supplementary material 1.

The control population comprised 17 healthy subjects (9 female/8 male), with a median age of 37 years and a range of 25-66, originating from the same geographical area as the patients.

All participants gave written informed consent, and the study was approved by the Institutional Local Research Bioethics Committee (Wroclaw Medical University—KB-321/2010).

### 2.2. Cell Isolation and Culture Conditions

Peripheral blood mononuclear cells (PBMCs) were isolated and cultured as described in supplementary material 2.

#### 2.2.1. mRNA Study

The subpopulations of T and B cells were separated from refrozen PBMC as described in supplementary material 1. Total RNA was extracted from 1 × 10^6^ cells [19], then 500 ng of RNA was reverse transcribed with the iScript cDNA Synthesis Kit (Bio-Rad, USA). The mRNA levels of BTLA and *β*2 microglobulin (*β*2M) as the reference gene were determined using commercial assays (Applied Biosystems, USA). All samples were assayed in duplicate using the 7300 Real-Time PCR System (Applied Biosystems, USA). The results were calculated according to the *Δ*CT method [20] by applying the *β*2M gene expression level as a reference.

#### 2.2.2. Immunostaining and Flow Cytometric Analysis

All experiments were carried out by triple labelling using the following monoclonal antibodies won from BD Biosciences (San Diego, USA): CD3-FITC, CD19-FITC, BTLA-PE, CTLA-4-PE-Cy.5, CD69-PE, and their appropriate isotype controls.

Surface staining of CD3, CD19, BTLA, and CTLA-4 was performed by standard protocols. Intracellular expression was determined only for the CTLA-4 protein in CD3+BTLA+ and CD19+BTLA+ cells. A detailed procedure of permeabilization and intracellular staining is described in supplementary material 1. After immunostaining, the cells were washed and analyzed by flow cytometry using a FACSCalibur cytometer (Becton Dickinson, BD Biosciences, San Diego, USA) equipped with CellQuest software for data analysis. At least 30,000 events per sample were analyzed in each experiment. The results were expressed as the proportion of CD3+ or CD19+ cells coexpressing BTLA and CTLA-4 molecules and as the mean fluorescence intensity (MFI) value expressed in arbitrary units (AU). Representative examples of flow cytometric analyses of the surface (s) and cytoplasmic (c) expression of CTLA-4 in CD19+BTLA+ cells and in CD3+BTLA+ are presented in supplementary materials 3 and 4, respectively.

#### 2.2.3. Statistical Analysis

Statistical analyses of the clinical data and laboratory findings were conducted using Statistica 10.0 software. For all analyzed variables, the median values and the 25^th^ and 75^th^ interquartile ranges were calculated. All collected data were examined for normal distribution using the Shapiro-Wilk test. For normally distributed data, the comparisons between studied groups were performed using the Student *t*-test for independent samples. In case of a nonnormal distribution, the Mann-Whitney *U* test for comparison between groups was used. To test the effects of stimulation, the Student *t*-test for dependent samples and the nonparametric Wilcoxon signed-rank test were applied. In all analyses, differences were considered significant when *p* ≤ 0.05.

## 3. Results

### 3.1. Expression of BTLA and CTLA-4 Immune Checkpoints in PB B and T Cells of CLL Patients and Healthy Controls

As was mentioned above, we and others have observed the involvement of the protein CTLA-4 expression in T and B in CLL immunopathology [5–8]. Moreover, we noticed that *BTLA* and *CTLA-4* gene polymorphisms are associated with mRNA expression and that variations in their genes might be considered as potential CLL risk factors [21, 22]. Therefore, we wanted to find out whether CLL development does affect BTLA and CTLA-4 suppressor expression at both the mRNA and the protein level in circulating B and T cells involved in systemic immunosuppression in CLL.

**Figure 1:**
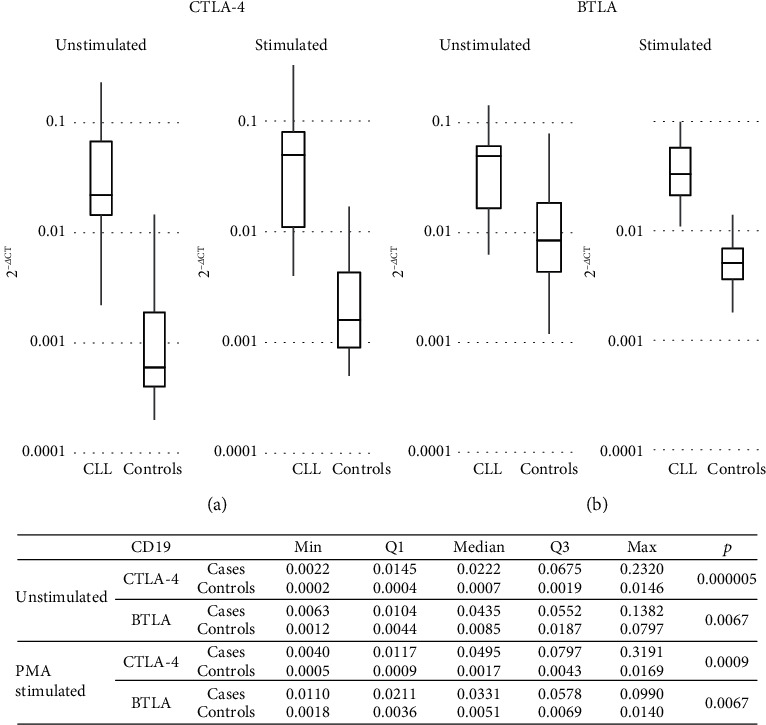
(a) CTLA-4 mRNA expression levels in CLL and controls in subpopulation of CD19-positive cells; (b) BTLA mRNA expression levels in CLL and controls in subpopulation of CD19-positive cells.

In freshly isolated B cells, BTLA and CTLA-4 mRNA expression levels were substantially higher in CLL patients than in healthy controls (HC) (*p* = 0.0067 and *p* = 0.000005, respectively; Figure 1).

**Figure 2:**
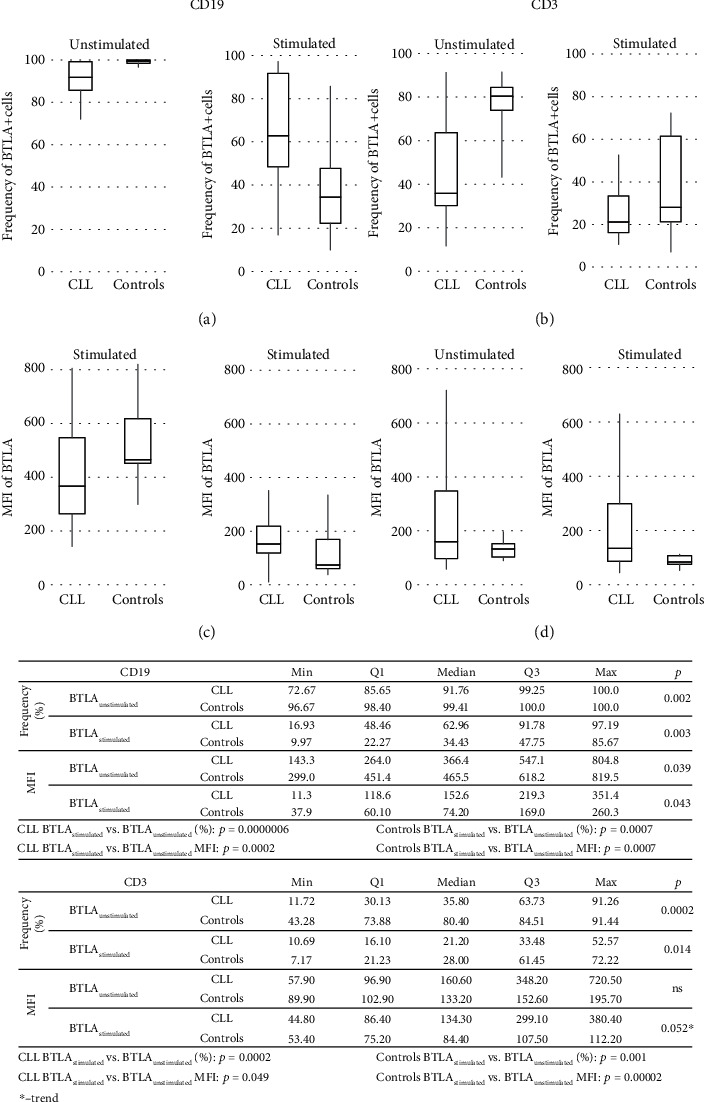
BTLA expression in B (CD19+) and T (CD3+) cell populations in unstimulated and stimulated cells from CLL patients and controls. The expression of BTLA on the surface of lymphocytes from patients and healthy donors was determined by flow cytometry. (a) The frequency of BTLA+ cells within circulating and in vitro stimulated CD19+ cells in CLL patients and controls is shown. (b) The frequency of BTLA+ cells within circulating and in vitro stimulated CD3+ cells in CLL patients and controls is shown. (c) The mean fluorescence intensity (MFI) of BTLA molecules on the surface of PB and in vitro stimulated CD19+ cells in CLL patients and controls is shown. (d) The mean fluorescence intensity (MFI) of BTLA molecules on the surface of PB and in vitro stimulated CD3+ cells in CLL patients and controls is shown. The horizontal lines show the median values, and the boxes exhibit interquartile ranges.

**Figure 3:**
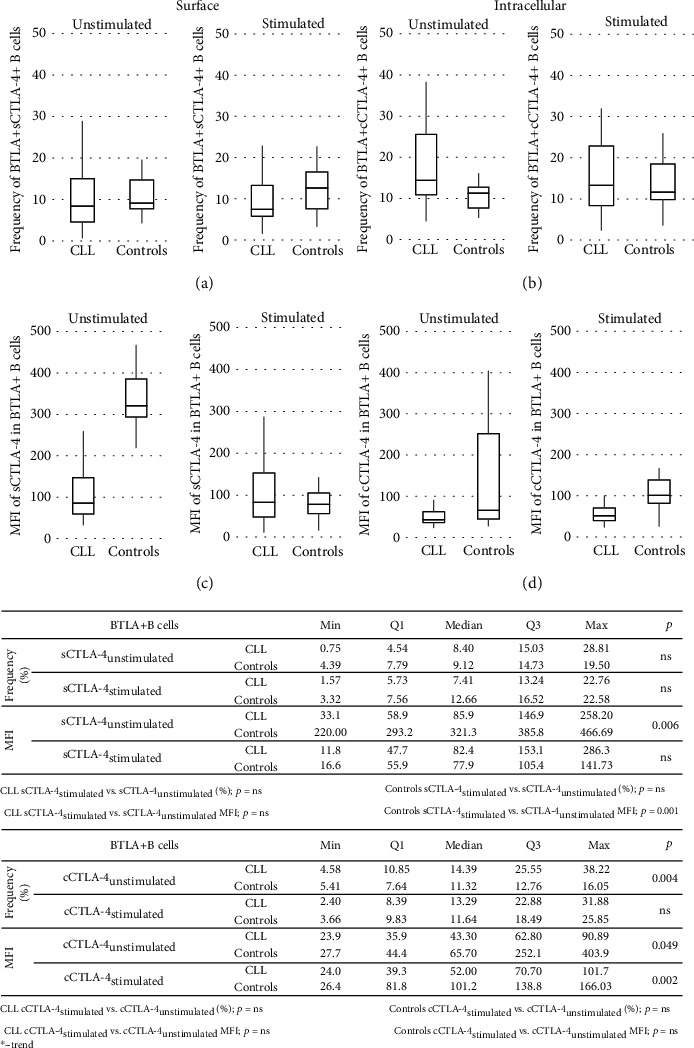
Surface and cytoplasmic CTLA-4 expression in unstimulated and stimulated BTLA+ B cells from CLL patients and controls. The expression of CTLA-4 and BTLA in lymphocytes from patients and healthy controls was determined by flow cytometry. (a) and (c) show the cell surface stained for both immune checkpoints, while (b) and (d) show the cell surface labeled with anti-BTLA mAb and then intracellularly labeled with anti-CTLA-4 mAb. (a) The frequency of BTLA+sCTLA-4+ cells within PB and in vitro stimulated CD19+ lymphocytes in CLL patients and controls is shown. (b) The frequency of BTLA+cCTLA-4+ cells within PB and in vitro stimulated CD19+ lymphocytes in CLL patients and controls is shown. (c) The mean fluorescence intensity (MFI) of CTLA-4 molecules on the surface (sCTLA-4) of PB and in vitro stimulated CD19+ cells in CLL patients and controls is shown. (d) The mean fluorescence intensity (MFI) of CTLA-4 molecules in the cytoplasm (cCTLA-4) of PB and in vitro stimulated CD19+ cells in CLL patients and controls is shown. The horizontal lines and the boxes show the median values and interquartile ranges, respectively.

At the protein level, mean fluorescence intensity (MFI) was significantly lower in the CLL group as compared to the controls for BTLA in B cells (*p* = 0.039, Figure 2) as well as CTLA-4 molecules (both surface and cytoplasmic) on/in a BTLA+ B cell subset (0.006 and 0.0049, respectively; Figure 3). In turn, qualitative analysis showed BTLA expression in almost all circulating B cells in the controls, while in the CLL cells, the median proportion of BTLA+ B cells was significantly lower, and in addition, the values were more diverse (*p* = 0.002; Figure 2). We also found a relatively low frequency of BTLA+ B cells coexpressing surface CTLA-4 (sCTLA-4) in both studied groups. In contrast, the higher proportion of BTLA-4+ B cells expressing cytoplasmic CTLA-4 (cCTLA-4) in CLL patients compared to the controls was noticed (*p* = 0.004; Figure 3).

**Figure 4:**
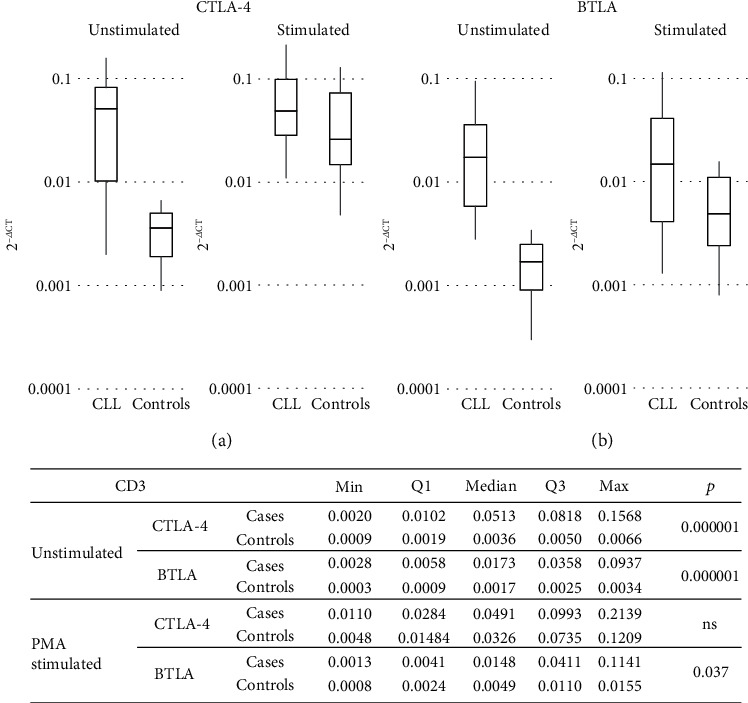
(a) CTLA-4 mRNA expression levels in CLL and controls in a subpopulation of CD3-positive cells; (b) BTLA mRNA expression levels in CLL and controls in a subpopulation of CD3-positive cells.

**Figure 5:**
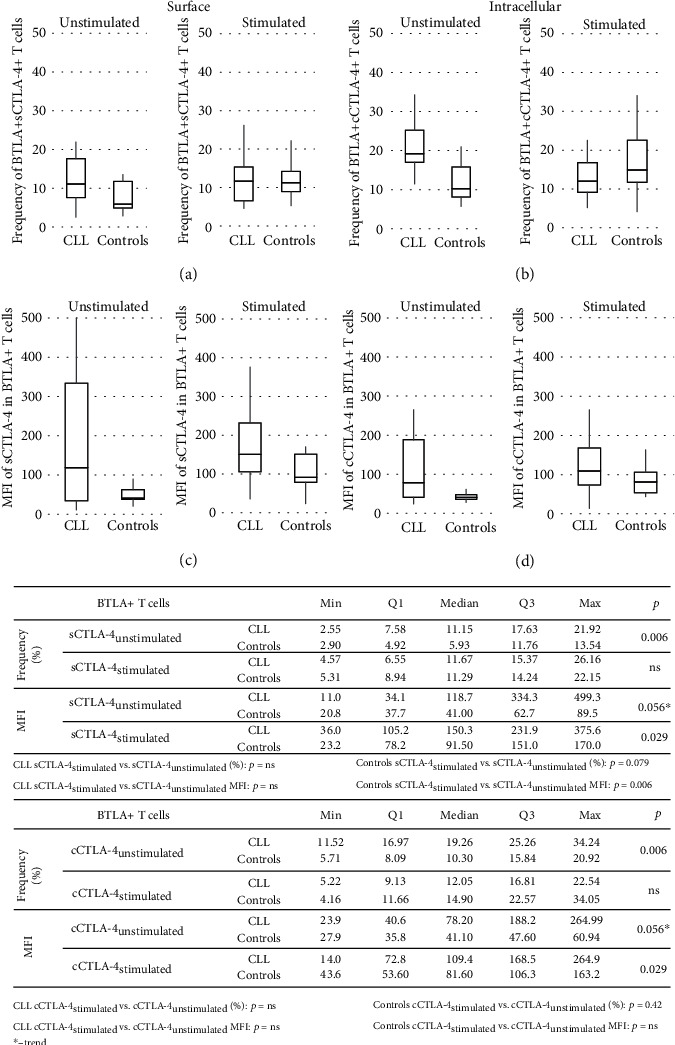
Surface and cytoplasmic CTLA-4 expression in unstimulated and stimulated BTLA+ T cells from CLL patients and controls. The expression of CTLA-4 and BTLA in lymphocytes from patients and healthy controls was determined by flow cytometry. (a) and (c) show the cell surface stained for both immune checkpoints, while (b) and (d) show the cell surface labeled with anti-BTLA mAb and then intracellularly labeled with anti-CTLA-4 mAb. (a) The frequency of BTLA+sCTLA-4+ cells within PB and in vitro stimulated CD3+ lymphocytes in CLL patients and controls is shown. (b) The frequency of BTLA+cCTLA-4+ cells within PB and in vitro stimulated CD3+ lymphocytes in CLL patients and controls is shown. (c) The mean fluorescence intensity (MFI) of CTLA-4 molecules on the surface (sCTLA-4) of PB and in vitro stimulated CD3+ cells in CLL patients and controls is shown. (d) The mean fluorescence intensity (MFI) of CTLA-4 molecules in the cytoplasm (cCTLA-4) of PB and in vitro stimulated CD3+ cells in CLL patients and controls is shown. The horizontal lines and the boxes show the median values and interquartile ranges, respectively.

In PB T cells, similar to B cells, whether for CTLA-4 or for BTLA, the median mRNA expression levels in the CLL patients were higher than those in the controls (*p* = 0.000001 and *p* = 0.000001, respectively; Figure 4). Although at the protein level, we noticed in CLL patients the increased amounts (measured by MFI) of BTLA in circulating T cells (*p* = ns; Figure 2) and trend for a higher expression of CTLA-4 (both sCTLA-4 and cCTLA-4) molecules on/in BTLA+ T cells, (*p* = 0.056 and *p* = 0.056, respectively; Figure 5).

In turn, an assessment of peripheral BTLA+ T cell distribution showed the significant lower frequency of these cells in CLL patients compared to the controls (*p* = 0.0002; Figure 2). Nevertheless, for BTLA+ T cells coexpressing sCTLA-4 or cCTLA-4 molecules, we found the significantly higher proportions in the CLL group as compared to the controls (*p* = 0.006 or *p* = 0.006, respectively; Figure 5).

From these results, it seems that CLL development strengthens *BTLA* and *CTLA-4* gene induction in peripheral B and T cells, while probably affecting the epigenetic modification of transcripts leading to a significant dysregulation of both checkpoint inhibitors' expression.

### 3.2. The Influence of *In Vitro* Stimulation on BTLA and CTLA-4 Expression in B and T Lymphocytes in CLL Patients

Circulating lymphocytes from CLL patients have recently been found to display activated and exhausted phenotypes characterized by, among others, the immune checkpoint expression [23]. In order to determine CLL B and T cell capacity for further activation and, in consequence, modulation of the inhibitory molecules BTLA and/or CTLA-4 expression, we performed a short-term stimulating culture of PBMCs. We used polyclonal stimulators to mimic the *in vivo* condition characterized by the presence of various microenvironmental factors. The obtained results have been compared to those seen in healthy controls.

PMA stimulation of PBMC revealed a statistically significant difference between the frequencies of both B and T cells expressing the CD69 activation marker in both groups studied; the CD69+ B cell compartment was higher than the CD69+ T cell subset (CLL: *p* = 0.0385 and HC: *p* = 0.0113; data not shown). However, comparative analysis performed between the groups showed that the markedly lower percentages of B and T cells from CLL patients responded to stimulation and expressed CD69 in the stimulating culture confronting the corresponding healthy cells (*p* = 0.00001 and *p* = 0.00036, respectively).

**Figure 6:**
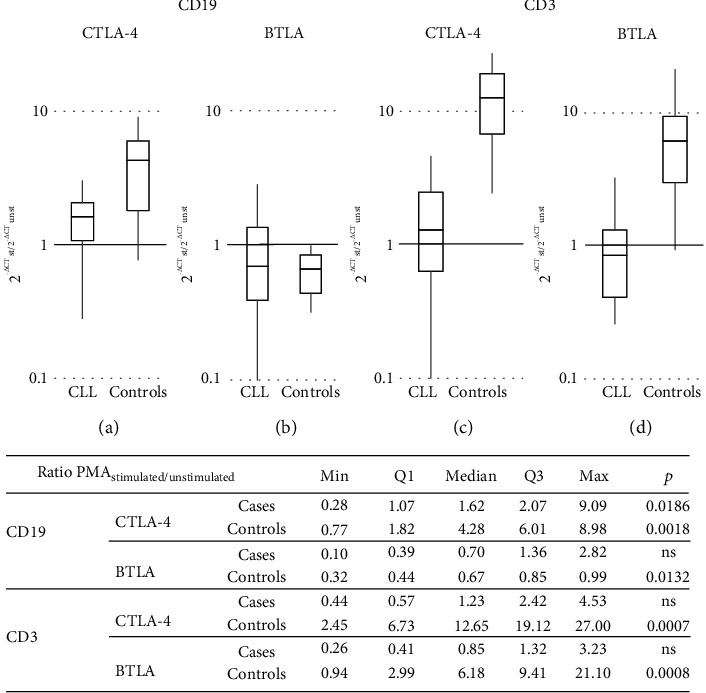
The effect of *in vitro* stimulation on mRNA expression levels for BTLA and CTLA-4 (demonstrated as the ratio (*R* = 2 − delta delta CT) of PMA-stimulated to unstimulated expression levels of CTLA-4 and BTLA). (a) Ratio of PMA-stimulated to unstimulated CTLA-4 mRNA expression levels in CD19 cells. (b) Ratio of PMA-stimulated to unstimulated BTLA mRNA expression levels in CD19 cells. (c) Ratio of PMA-stimulated to unstimulated CTLA-4 mRNA expression levels in CD3 cells. (d) Ratio of PMA-stimulated to unstimulated BTLA mRNA expression levels in CD3 cells.

In the B cell population, compared to the prestimulation level, we observed a PMA-induced significant increase in the CTLA-4 mRNA expression in both studied groups, although more pronounced in the controls (*p* = 0.0186 and *p* = 0.0018, respectively; Figures 1 and 6).

In contrast, *in vitro* stimulation caused the 1.5-fold reduction in BTLA mRNA levels both in CLL patients and the controls; however, the changes seen in CLL were not significant (*p* = ns and *p* = 0.0132, respectively). Thereby, patients' *in vitro* stimulated B cells maintained a substantially higher expression of mRNA for both CTLA-4 and BTLA compared to the controls (*p* = 0.0009 and *p* = 0067, respectively; Figure 1). Consistently, the *in vitro* stimulation induced a significant decrease of BTLA protein expression in all individuals when measured either as cell frequency or as fluorescence intensity. Remarkably, its reduction was more pronounced in healthy B cells, whereby patients remained with a significantly higher expression of the BTLA molecule in these stimulated cells (for all comparisons, *p* ≤ 0.007; Figure 2).

In a subset of BTLA+ B cells coexpressing the CTLA-4 molecule, we showed in healthy cells a stimulated decrease in the fluorescence intensity of sCTLA-4 (*p* = 0.001) and no changes in the level of the cCTLA-4 molecule (Figure 3). In CLL, there were no significant differences in the amounts measured by MFI of CTLA-4 molecules (both sCTLA-4 and cCTLA-4) in stimulated BTLA+ B cells in comparison with baseline levels. Therefore, after stimulating the culture, sCTLA-4 intensity values in BTLA+ B cells were similar in both studied groups, whereas the cCTLA-4 MFI level was lower in CLL (*p* = 0.049; Figure 3).

Similarly, we found that the only qualitative change in the cultured B cell subtypes in response to stimulation was assigned to the healthy BTLA+ B cells. Within these cells, we noticed a significant increase in the frequency of cCTLA-4+ cells (*p* = 0.042; Figure 3), while the percentages of stimulated sCTLA-4+ cells remained unchanged. In addition, no changes were found within the BTLA+ B cells expressing CTLA-4 (both sCTLA-4 and cCTLA-4) from CLL patients during stimulation. In consequence, the median proportions of *in vitro* activated BTLA+ B cells with CTLA-4 expression did not differ significantly between the CLL and control groups (Figure 3).

Regarding the stimulation-induced differences in the *BTLA* and *CTLA-4* gene expression within T cells, no significant influence has been noted in CLL (Figure 4). In contrast, the total stimulated mRNA expression for both inhibitory genes in the healthy T cell population changed significantly; the CTLA-4 mRNA expression increased more than 12-fold, while BTLA mRNA levels increased more than sixfold (*p* = 0.0007 and *p* = 0.0008, respectively; Figure 6). In consequence, the mRNA for CTLA-4 reached comparable levels in both studied groups, whereas BTLA gene transcripts still remained upregulated in patients (*p* = 0.037; Figure 4).

When we analyzed the MFI of BTLA molecules in PMA-stimulated T cells, we found its values to be higher in the CLL group than in the controls (*p* = 0.052), suggesting that the stimulated decrease in BTLA level was more pronounced in healthy donors (Figure 2).

Considering the CTLA-4 expression in a subset of stimulated BTLA+ T cells, we noticed a twofold increase in the MFI of sCTLA-4 and cCTLA-4 in T cells from the controls compared to the prestimulation levels (*p* = 0.006 and *p* = 0.002, respectively; Figure 5). In contrast, *in vitro* stimulation resulted in no significant differences in the intensity of both CTLA-4 molecules in corresponding cells in CLL patients. Nevertheless, we observed the maintenance of higher MFI values of both sCTLA-4 and cCTLA-4 in stimulated BTLA+ T cells in CLL patients compared to the controls (both *p* = 0.029; Figure 5).

In the qualitative analysis, a similar pattern of T cell response to stimulation was observed in both studied groups when considering the BTLA expression. Although a substantial decrease in the BTLA+ T cell frequencies in both studied groups was noticed (CLL: *p* = 0.0002 and HC: *p* = 0.001; Figure 2), a prevalence of those subsets was sustained in the controls (*p* = 0.014; Figure 2).

For a subset of stimulated BTLA+ T cells coexpressing sCTLA-4 or cCTLA-4 molecules, the median proportions of those cells were similar in both groups due to an increase in sCTLA-4 abundance in the controls (*p* = 0.079) and a decrease in cCTLA-4 in the CLL patients (*p* = 0.006; Figure 5).

Summarizing the above results, there are substantial differences in the pattern of response to *in vitro* stimulation between CLL patients and healthy subjects, suggesting hyporesponsiveness of CLL circulating lymphocytes, primarily T cells.

## 4. Discussion

The expression of the immune checkpoint inhibitors in CLL has been the subject of several studies [2, 4, 6, 7, 16, 24]. In our previous report, we showed the increased frequency of circulating CTLA-4+ T cell subtypes as well as the disturbed kinetics of its expression, suggesting a suppressed T cell-mediated immunity in CLL [2]. In addition, we observed that heightened proportions of CLL cells expressing surface and cytoplasmic CTLA-4 negatively correlated with disease progression [7]. Furthermore, our recent study on the blocking of the CTLA-4 molecule on CLL cells strongly demonstrated its opposite effects on CLL cells and its dependency on the expression levels of CTLA-4 on leukemic cells. For some patients, systemic administration of anti-CTLA-4 monoclonal antibody might be an unfavourable immunotherapeutic strategy, as CTLA-4 blocking might induce prosurvival signals in the high CTLA-4 expressers [8].

The present study has extended our research on the potential involvement of BTLA and CTLA-4 checkpoint inhibitors expressed in circulating lymphocytes in the pathogenesis of CLL. For this purpose, we determined BTLA and CTLA expression at the mRNA and the protein level in T and B lymphocytes from PB of CLL patents in relation to results obtained for controls. Herein, we showed that in PBMCs of CLL patients, both CTLA-4 and BTLA suppressors were overexpressed at the mRNA level either in the T or B cell subpopulation. Surprisingly, although we observed the higher mRNA of BTLA in CLL, its expression at the protein level was not upregulated in circulating lymphocytes and was even lower in B cells. The mechanisms underlying the observed defect in BTLA expression in CLL are not clearly understood. Taking into account that we did not find any defect in *BTLA* gene transcription and augmented levels of *BTLA* transcripts were seen in CLL samples, the alterations in posttranslational control should rather be considered as a cause of the impairment of BTLA protein expression in CLL. One of the reported mechanisms of epigenetic modifications has been assigned to miRNA regulation of the protein expression. In fact, it was recently shown that BTLA gene expression can be posttranslationally regulated by miRNA-155 during naïve T cell activation [25]. The epigenetic modification involvement in the pathogenesis of CLL is well established [26–28]. Many miRs are considered as factors influencing CLL risk and prognosis, among others miR-155-5p. The expression of this miR is upregulated and associated with higher disease risk and poorer prognosis [29–31]. The influence of miRs' epigenetic regulation of BTLA protein expression is the subject of our ongoing study.

Although we found that BTLA was expressed in a relatively high proportion of B cells and was less pronounced in T cells in all subjects, in CLL patients, BTLA+ cell frequency was significantly decreased.

Studies on the expression of BTLA in hematological malignancies are very limited. M'Hidi et al. [16] investigated the *in vivo* distribution of BTLA within human normal lymph nodes. Moreover, they showed BTLA expression in CLL/small lymphocytic lymphoma (B-CLL/SLL), but not in other B cell lymphomas including follicular lymphoma, mantle cell lymphoma, and marginal zone lymphoma. Further studies on lymphoma cells indicated that the BTLA-HVEM pathway participates in the differentiation and inhibition of *γδ* T cells after exposure to lymphoma cells [32], suggesting its involvement in lymphomagenesis. In recent work, Kang et al. [33] investigated associations of the expression of immune checkpoint molecules, among others BTLA and HVEM, with prognosis in childhood acute leukemia (AML and ALL). They found a similar expression of BTLA on *γδ*+ T and *αβ*+ T cells from ALL and AML patients as well as healthy controls; however, they noticed a significantly higher expression of HVEM on *αβ*+ T cells in the low-risk group of AML patients as compared to the high-risk group. The high expression of BTLA was found in B cell lines, primarily on the multiple myeloma cells [34].

In turn, in healthy individuals, the expression of BTLA in T cells was recently described by Otsuki et al. [35], similar to us, showed the expression of BTLA on approximately 90% of T cells and B cells within freshly isolated PBMCs.

The lower expression of BTLA in patient lymphocytes found in our study may reflect the state of systemic activation in CLL by microenvironmental factors, such as neoplastic antigens and cytokines, and is consistent with other reports showing that BTLA is gradually decreased during stimulation [32, 35]. This seems reasonable since BTLA is a suppressor of immune response initiation [32]. The increased frequency of CLL BTLA+ cells coexpressing CTLA-4, the expression of which is inducible upon cell stimulation [36, 37], additionally strengthens the above suggestion of the systemic activation in CLL. Moreover, we observed that *in vitro* stimulation led to further BTLA expression decrease in B and T cells in all studied individuals. It is worthy to note, however, that the decrease was pronounced in healthy controls, which is in agreement with other observations [35]. Additionally, stimulation with PMA changed the CTLA-4 protein expression in healthy lymphocytes compared to a lack of such a response in CLL; the significant increase in the levels of this protein has been demonstrated (primarily in T cell populations) and is consistent with the well-known impact of activation on *CTLA-4* gene induction [37]. Therefore, in contrast to lymphocytes from CLL patients, those of healthy subjects exhibited an unstimulated phenotype and possess a potency for optimal activation under environmental stimulating conditions. The level and pattern of *in vitro* activated expression of CD69, BTLA, and CTLA-4 indicate not only hyporesponsiveness of PB lymphocytes from CLL patients, primarily T cells, but also their exhausted phenotype. Consistently, former studies have described the influence of the tumor antigen load in the microenvironment on the lymphocyte exhaustion and dysfunction [38, 39].

It has been reported that an impairment of BTLA expression in normal and lymphoma B cells may lead to cell-autonomous BCR activation and induce B cell growth, as a loss of BTLA dampers the threshold for cell activation [32]. Consistently, it has been demonstrated that CLL cells express the phenotype of activated B lymphocytes, resembling cells undergoing chronic antigenic stimulation [40]. Worthy of note is also our original observation on the diminished level of surface expression of CTLA-4, which is a functionally suppressive molecule acting primarily in the late phase of stimulation, which was in strike contrast to the T cell compartment. This finding suggests that *in vivo* activated B cells in CLL have a lower potential to efficiently terminate the ongoing B cell activation, thereby proliferating at a higher rate in proliferative centers of secondary lymphoid tissue and, to a lesser degree, in PB [41, 42] and/or inducing a tumor-supportive microenvironment [43, 44].

The current study showed, in addition, that within peripheral lymphocytes from patients, the T cell compartment seems to be more suppressed under stimulating conditions. The qualitative and quantitative upregulation of the expression of the CTLA-4 molecule (both surface and cytoplasmic) observed by us in peripheral BTLA+ T cells in CLL may reflect a powerful suppression of T cell effector functions, including antitumor and anti-inflammatory activities. The significantly lower proportion of CLL T cells expressing the activation marker CD69 in the stimulation culture seems to confirm substantial hyporesponsiveness of this cell population. T cell-mediated immunosuppression is of clinical relevance and plays an important role in CLL progression and/or clinical complications [45–48]. Further functional studies are needed to confirm the significance of our data indicating the involvement of the dysregulated expression of both BTLA and CTLA-4 checkpoint inhibitors in PB lymphocytes in CLL immunopathology. Such experiments are currently being performed in our lab to verify the above suggestions.

## 5. Conclusion

We found for the first time the dysregulated expression of both BTLA and CTLA-4 suppressors in B and T lymphocyte compartments of CLL patients, which may play a role in CLL immunopathology and clinical complications. The defected expression of BTLA, together with insufficient levels of CTLA-4 on CLL B cells, might be associated with lowering of the threshold for B cell activation and proliferation. On the other hand, upregulation of the expression of the CTLA-4 molecule (both surface and cytoplasmic) found in CLL peripheral BTLA+ T cells may affect T cell effector functions.

## Data Availability

The data used to support the findings of this study are available from the corresponding author upon request.

## Supplementary Materials

Supplementary 1 Clinical characteristics of CLL patients.

Supplementary 2 Detailed procedure of (a) cell isolation and stimulation, (b) permeabilization, and (c) T and B cell separation.

Supplementary 3 Representative examples of flow cytometric analyses of the surface (s) and cytoplasmic (c) expression of CTLA-4 in CD3+BTLA+ cells in studied groups of CLL patients and healthy controls before (A) and after cell culture under stimulating conditions (PMA+ionomycin) (B). Numbers on dot plots represent the percentage of CD3+ cells expressing BTLA. Gray histograms represent isotype controls. Numbers on histograms represent the percentage of CD3+BTLA+ cells expressing CTLA-4.

Supplementary 4 Representative examples of flow cytometric analyses of the surface (s) and cytoplasmic (c) expression of CTLA-4 in CD19+BTLA+ cells in studied groups of CLL patients and healthy controls before (A) and after cell culture under stimulating conditions (PMA+ionomycin) (B). Numbers on dot plots represent the percentage of CD19+ cells expressing BTLA. Gray histograms represent isotype controls. Numbers on histograms represent the percentage of CD19+BTLA+ cells expressing CTLA-4.
