# Supplementary material for: Abnormal Expression of BTLA and CTLA-4 Immune Checkpoint Molecules in Chronic Lymphocytic Leukemia Patients
Source: J Immunol Res. 2020 Jul 28;2020:6545921. doi: 10.1155/2020/6545921 (PMC7407019; doi:10.1155/2020/6545921)
Supplement: Supplementary 1 — Clinical characteristics of CLL patients. [file 6545921.f1.docx]

Supplementary material 1. Clinical characteristics of CLL patients

| Characteristics Value | | |
| --- | --- | --- |
| Number of patients |  | 21 |
| Gender(female/male) |  | 10/11 |
| Age (years, mean and SD) |  | 67.4±8.3 |
| Rai Stage |  |  |
|  | 0 | 7 |
|  | 1 | 6 |
|  | 2 | 4 |
|  | 3 | 0 |
|  | 4 | 4 |
| WBC count (1 x10^9^/l) |  | 39.7±27.3 |
| Lymphocyte count (1x 10^9^/l) |  | 38.4±28.0 |
| Hb level (g/dl) |  | 13.5±1.1 |
| Platelet count (1x 10^9^/l) |  | 170.0±48.5 |
| LDH (U/l) |  | 358.6±195.3 |
| b2-microglobulin (mg/l) |  | 4.0+2.8 |

For age and clinical parameters, the mean values and standard deviation

(SD) were presented
