# Supplementary material for: Abnormal Expression of BTLA and CTLA-4 Immune Checkpoint Molecules in Chronic Lymphocytic Leukemia Patients
Source: J Immunol Res. 2020 Jul 28;2020:6545921. doi: 10.1155/2020/6545921 (PMC7407019; doi:10.1155/2020/6545921)
Supplement: Supplementary 2 — Detailed procedure of (a) cell isolation and stimulation, (b) permeabilization, and (c) T and B cell separation. [file 6545921.f2.doc]

Isolation and stimulation:

Peripheral blood mononuclear cells (PBMCs) were isolated by Lymphoflot (Bio-Rad Medical Diagnostics GmbH, Dreieich, Germany) density gradient centrifugation from anticoagulated venous blood samples of patients with CLL and healthy volunteers and then stored in liquid nitrogen. After thawing, PBMCs were suspended at 1x10^6^ PBMCs ml-1 in RPMI 1640 medium (Gibco, Paisley, UK) supplemented with 10% fetal calf serum (Flow Labs, UK), L-glutamine, and 50 g/ml gentamycin (Gibco, Paisley, UK) and cultured with 25 ng/ml phorbol 12-myristate 23-acetate (PMA) and 1 μg/ml of ionomycin (Ion) (Sigma-Aldrich, St. Gallen, Switzerland) in the presence of 10 μg/ml of brefeldin A (BFA, protein transport inhibitor) for 4 h at 37°C in a humidified atmosphere containing 5% CO_2_.

Permeabilization:

To perform permeabilization, the cells were fixed with 2% paraformaldehyde (Fluka, Sigma-Aldrich, Buchs, Germany), washed twice using PBS without Ca^++^ and Mg^++^ and subsequently permeabilized in BD-FACS Permeabilizing Solution 2 (BD Biosciences, San Diego, USA) according to the manufacturer’s instructions. The efficacy of permeabilization was determined by uptake of trypan blue. Following the permeabilization, the cells were incubated for 30 min at 4°C with anti-CTLA-4-PE-Cy.5 monoclonal antibody. Isotype-matched control antibodies were used in all experiments to confirm expression specificity.

T and B cell separation:

The subpopulations of T and B cells were separated from refrozen PBMC cells as described in supplementary material 1. with two steps: the first step employed the positive selection of T cells (CD3 positive cells) and was performed with a Human CD3 Selection Cocktail (StemCell Technologies Inc, Vancouver, Canada); the second step involved the negative selection of B cells in the remaining material and was done with the use of a Human B Cell Enrichment Kit without CD43 depletion (StemCell Technologies Inc, Vancouver, Canada) in CLL cells or with a Human B Cell Enrichment Kit (StemCell Technologies Inc, Vancouver, Canada) in the controls.
