# Supplementary figures and images for: Abnormal Expression of BTLA and CTLA-4 Immune Checkpoint Molecules in Chronic Lymphocytic Leukemia Patients
Source: J Immunol Res. 2020 Jul 28;2020:6545921. doi: 10.1155/2020/6545921 (PMC7407019; doi:10.1155/2020/6545921)

**A**

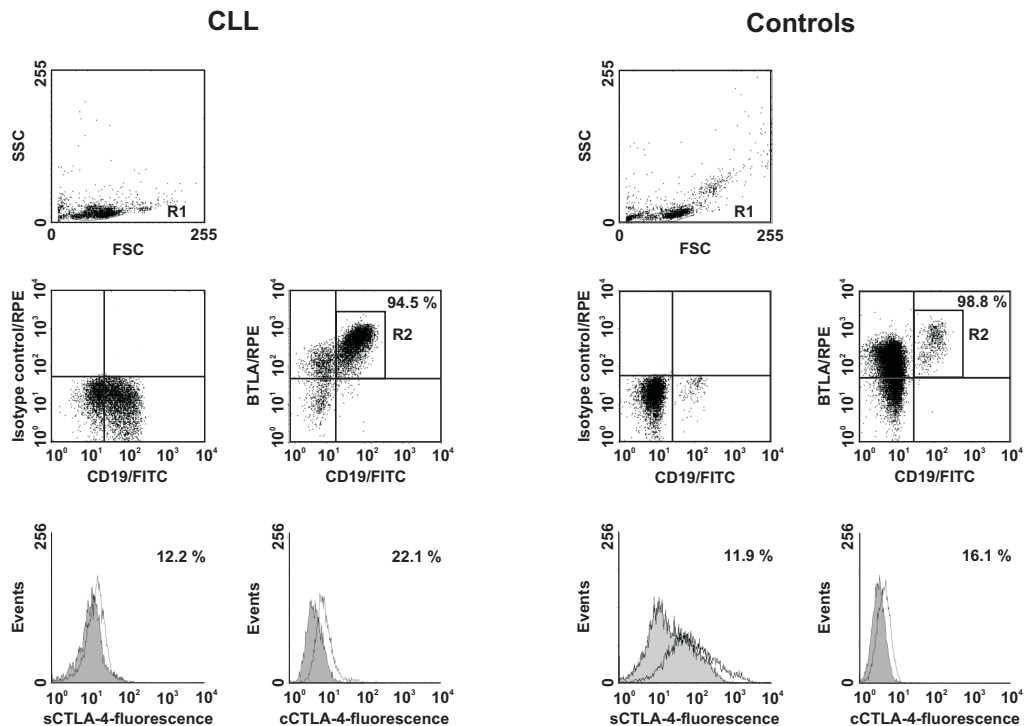

**B**

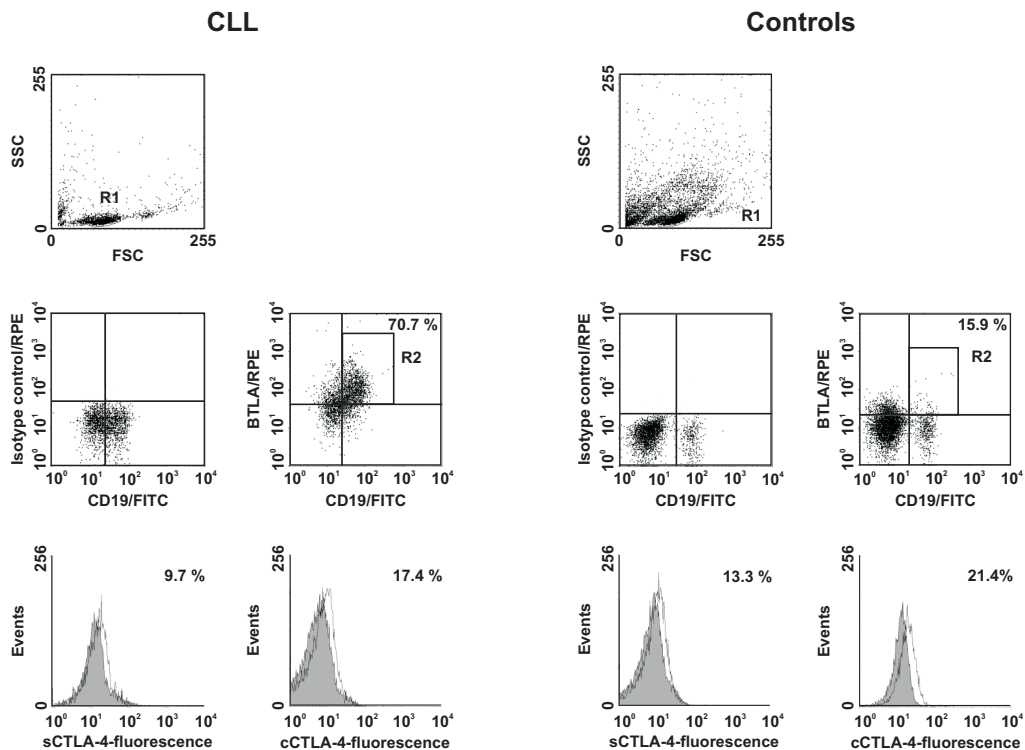

Supplement: Supplementary 3 — Representative examples of flow cytometric analyses of the surface (s) and cytoplasmic (c) expression of CTLA-4 in CD3+BTLA+ cells in studied groups of CLL patients and healthy controls before (A) and after cell culture under stimulating conditions (PMA+ionomycin) (B). Numbers on dot plots represent the percentage of CD3+ cells expressing BTLA. Gray histograms represent isotype controls. Numbers on histograms represent the percentage of CD3+BTLA+ cells expressing CTLA-4. [file 6545921.f3.pdf]

A

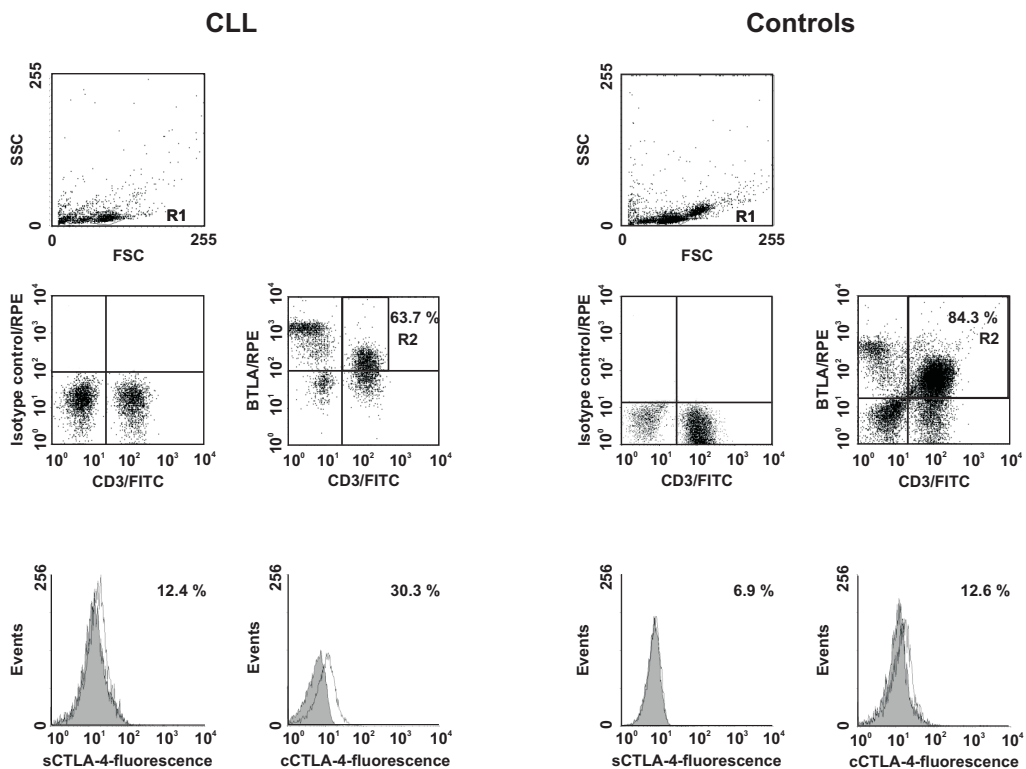

B

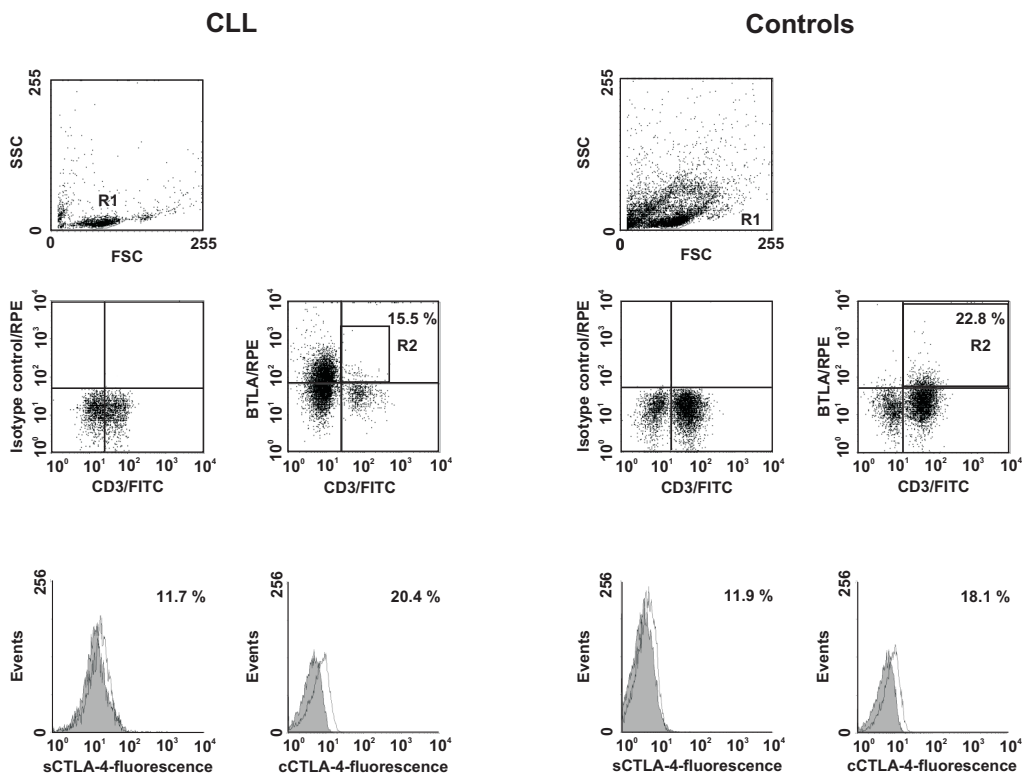

Supplement: Supplementary 4 — Representative examples of flow cytometric analyses of the surface (s) and cytoplasmic (c) expression of CTLA-4 in CD19+BTLA+ cells in studied groups of CLL patients and healthy controls before (A) and after cell culture under stimulating conditions (PMA+ionomycin) (B). Numbers on dot plots represent the percentage of CD19+ cells expressing BTLA. Gray histograms represent isotype controls. Numbers on histograms represent the percentage of CD19+BTLA+ cells expressing CTLA-4. [file 6545921.f4.pdf]
